# Gelatin Blends Enhance Performance of Electrospun Polymeric Scaffolds in Comparison to Coating Protocols

**DOI:** 10.3390/polym14071311

**Published:** 2022-03-24

**Authors:** Maria Bikuna-Izagirre, Javier Aldazabal, Jacobo Paredes

**Affiliations:** 1 Tecnun School of Engineering, University of Navarra, Manuel Lardizabal 13, 20018 San Sebastian, Spain; mbikunai@tecnun.es (M.B.-I.); jaldazabal@tecnun.es (J.A.); 2 Biomedical Engineering Centre, University of Navarra, Campus Universitario, 31080 Pamplona, Spain

**Keywords:** electrospinning, scaffold, PCL, gelatin, tissue engineering, mechanical properties, characterization

## Abstract

The electrospinning of hybrid polymers is a versatile fabrication technique which takes advantage of the biological properties of natural polymers and the mechanical properties of synthetic polymers. However, the literature is scarce when it comes to comparisons of blends regarding coatings and the improvements offered thereby in terms of cellular performance. To address this, in the present study, nanofibrous electrospun scaffolds of polycaprolactone (PCL), their coating and their blend with gelatin were compared. The morphology of nanofibrous scaffolds was analyzed under field emission scanning electron microscopy (FE-SEM), indicating the influence of the presence of gelatin. The scaffolds were mechanically tested with tensile tests; PCL and PCL gelatin coated scaffolds showed higher elastic moduli than PCL/gelatin meshes. Viability of mouse embryonic fibroblasts (MEF) was evaluated by MTT assay, and cell proliferation on the scaffold was confirmed by fluorescence staining. The positive results of the MTT assay and cell growth indicated that the scaffolds of PCL/gelatin excelled in comparison to other scaffolds, and may serve as good candidates for tissue engineering applications.

## 1. Introduction

Electrospinning is a simple, low-cost and versatile method for the production of polymeric nanofibrous scaffolds. These mesh structures offer high porosity and high surface area, thereby enhancing cell behavior, i.e., adhesion, proliferation, etc. [1,2,3]. Such characteristics are key for scaffold designs, and can be modulated by the changing fabrication parameters or selecting a specific polymer [4,5].

A broad range of materials can be electrospun into nanofibers. Synthetic polymers have been widely used for different tissue engineering (TE) applications such as bone and cardiac tissue with poly-L-lactide (PLLA) [6,7], skin regeneration based on poly(lacti acid-co-glycolic acid) (PLGA) [7], soft tissue applications with poly(ethylene oxide) (PEO) [8] and wound healing assays with polyvinyl alcohol (PVA) [9]. In addition, polycaprolactone (PCL) has been used in several studies due to its desirable properties. For instance, this semicrystalline polymer offers lower degradation rates (1 to 2 years) than poly(glycolide) or poly(lactide) [10], which is an interesting feature for long-term implants. Moreover, it shows high tensile strength (10.5–16.1 MPa), yield strength (8.2–10.1 MPa) and elastic modulus (343.9–364.3 MPa) [11,12]. The mechanical characteristics and biodegradability rates [13] of synthetic polymers, which are critical features in scaffold design, can be adjusted for each clinical application [4,14]. Generally, the ideal scaffolds for tissue regeneration should possess good biocompatibility, biodegradability, easy fabrication, and sufficient mechanical properties. The modified PCL nanofibers fulfill these requirements and have been shown to be suitable for sutures, tendons, cartilage, bone [15], nerve [16] and other TE applications [15,17].

Unfortunately, despite the widespread use of synthetic polymers in the field of TE, these materials are not without their faults. Among their most significant drawbacks is the high hydrophobicity of the raw materials used to create these polymers, which hinders cellular attachment and scaffold infiltration, and ultimately, constrains cellular proliferation [18].

The aforementioned drawback, which is inherent to the surface interaction of synthetic materials, can be overcome via two different approaches: oxygen plasma treatment, that produces changes in the chemical groups of the surface, thereby improving the wettability [18,19]; or the use of natural polymers either as a coating of the synthetic materials [20,21], or by mixing both in a blend [22,23]. Natural polymers, such as collagen, gelatin, chitosan, elastin, fibrinogen and laminin, provide a hydrophilic environment and contain integrin-binding sites for the cell-surface receptors that facilitate cell adhesion [24]. However, despite their suitability, natural polymers alone usually have poor mechanical properties, rapid biodegradability rates, and manipulation/fabrication issues [3].

When they are applied as fiber coatings over a synthetic polymer, natural polymers cause an almost negligible change in the mechanical performance of the final mesh, and bring about improvements in its surface properties [20]. However, as the natural coatings are typically uncrosslinked to the synthetic polymer, they dissolve rapidly and their bioactivity is rapidly lost [23,25]. In contrast, when mixing natural polymers into a blend, we can not only provide the appropriate surface characteristics, but we can also modify the mechanical properties of the final mesh, which is difficult to achieve using coatings [25]. Moreover, the longer-lasting biocompatibility of scaffolds built via blending [26,27] compared to coated scaffolds makes them useful in many of the aforementioned TE applications [14,15,28]. In fact, PCL/gelatin composites have been shown to achieve a reduction in the Young Modulus of the final mesh, which improves the scaffold’s output in nerve regeneration [29], cartilage regeneration [30], retinal tissue engineering [31] and bone regeneration membranes [23]. PCL/gelatin scaffolds have also shown great potential for wound healing and skin regeneration applications [32,33].

Both blending and coating are known to enhance scaffold outcomes in terms of cellular fate [20,21,22,23]. However, despite their widespread application in the field of TE, the literature is scarce when it comes to comparing the performance of nanofibrous scaffolds constructed based on these two methods. At the time of writing and to the best of our knowledge, no studies to date have compared the benefits and drawbacks of these scaffold construction strategies. To address this, in this work, we begin by constructing PCL scaffolds using both the coating and blending methods. Then, we study how the mechanical properties of these scaffolds change based on how they have been built (either by coating or blending). Finally, we assess the impact that these methodologies can have on cell performance by analyzing the change of mouse embryonic fibroblast (MEF) behavior when these cells are placed on the PCL scaffolds built for this study.

## 2. Materials and Methods

### 2.1. Materials and Reagents

PCL pellets (Mw = 800,000) and gelatin powder of porcine skin were purchased from Sigma-Aldrich (St. Louis, MO, USA). Glacial acetic acid and chloroform were purchased from Sigma-Aldrich (St. Louis, MO, USA), and methanol from AppliChem Panreac (Barcelona, Spain) and 3-(4,5-dimethylthiazol-2-yl-2,5-diphenyltetrazolium bromide) (MTT) were obtained from Roche.

### 2.2. Fabrication of Nanofiber Scaffolds

For electrospinning, first, PCL (10 wt.%) and PCL (20 wt.%) were added to chloroform/methanol (3:1, *v/v*), agitating the mixtures with a magnetic stirrer at 600 rpm overnight at room temperature (23 ± 1 °C) [34]. Gelatin (8 wt.%) was dissolved in acetic acid (80% *v/v*) by stirring the mixture vigorously for 3 h at room temperature. After the preparation of the polymeric solution, PCL (20 wt.%)/gelatin at 80:20 volume ratio was mixed and stirred for 48 h.

**Figure 1:**
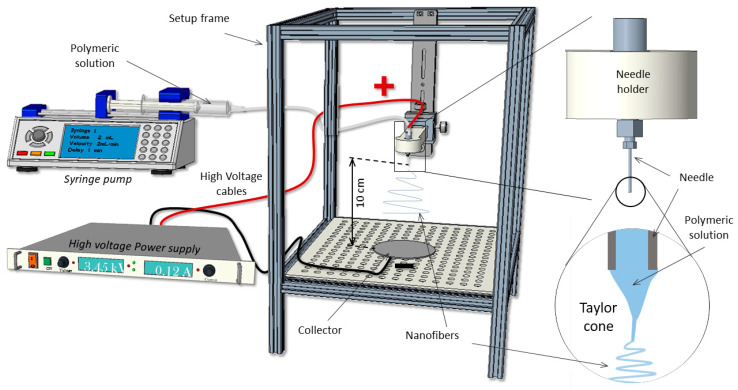
Home-made electrospinning setup.

Nanofiber membranes were fabricated on an upright, home-made setup (Figure 1), using a controlled flow rate (0.5 mL/h) with a syringe pump (Chemyx Fusion 100, Stafford, TX, USA) which was connected to a blunt metallic 20 G needle through a capillary Teflon^®^ tube. A high voltage DC power supply (FC Series 120 watt, CE Compliant) was used to provide the necessary electric field between the needle and the collector.

For the process of electrospinning, solutions were placed in a 1 mL plastic syringe. For PCL (10 wt.%), a voltage of 13 kV was applied, with a distance of 10 cm between needle and collector. The blended PCL (20 wt.%)/gelatin solution was extruded under 22 kV and a distance of 10 cm. The nanofibers were collected on a 9 × 9 cm^2^ piece of flat aluminum foil.

### 2.3. Characterization of Nanofibrous Scaffold

The morphology of the nanofibrous scaffolds was studied with FE-SEM (Zeiss, Gemini, Germany) at an accelerating voltage of 5 kV. Fiber diameters and pore areas of the scaffolds were calculated on the basis on FE-SEM images by using image analysis software (Image J, NIH, Madison, WI, USA) and the Diameter J plugin (Nathan Hotaling—v1.018), respectively.

Mechanical measurements were obtained by applying tensile test loads to specimens prepared from the electrospun nanofibrous mats. In this study, five specimens from each condition were cut with a caliper to widths of 11 mm. Once the samples (aluminum foil and the nanofibers) had been cut with a microtome blade, the electrospun mats were detached with tweezers from the foil and placed on the traction testing machine (ZwickiLine Z1.0, Zwick/Roell, Ulm, Germany) with a load cell of 50 N (Xforce P, Zwick/Roell, Germany) at room temperature. The initial distance between grips was 10 mm (studied area was 11 × 10 mm^2^), and the crosshead displacement speed was set to 100 mm/min for all tests. The thickness was measured with a thickness digital gauge (Digimatic Serie 547, Mitutoyo, Kana, Japan).

FT-IR spectroscopy of PCL, gelatin coated PCL and blended PCL/gelatin mats were recorded in triplicate over the range of 4000 cm^−1^ and 400 cm^−1^. The gelatin was dissolved in deionized water and later added to the PCL electrospun nanofiber. The blended PCL/gelatin samples were fabricated with the aforementioned volume ratios. All samples were dried under vacuum for 24 h before analysis.

### 2.4. Cell Culture

Mouse embryonic fibroblasts (MEFs) [35] were cultured as described in [36], using Dulbecco’s modified eagle medium (DMEM, ThermoFisher Scientific, Waltham, MA, USA) supplemented with 10% FBS (ThermoFisher Scientific) and 1% penicillin—streptomycin (ThermoFisher Scientific), and 1.5% HEPES 1 M (Sigma Aldrich, St. Louis, MO, USA).

### 2.5. Cell Viability Assay

The viability of MEF cells on nanofibrous scaffolds was quantitatively determined using colorimetric MTT assay. Before cell seeding, scaffolds were treated with a plasma (100 W for 1 min, with 5 and 15 sccm for O_2_ and Ar respectively) process (Diener Electronic, Ebhausen, Germany) in order to increase the hydrophilicity of the surface. Then, electrospun meshes were placed on 24 well plate cell crowns (Scaffdex, Tampere, Finland) and sterilized with UV radiation from both sides for 30 min each. Subsequently, the scaffolds were incubated in a 24 well tissue culture plate with MEF cells at a density of 2 × 10^4^ cell per well for 24, 48 and 72 h at 37 °C in 5% CO_2_ incubator. After the incubation period, the wells were washed with DPBS, and then 50 μL of MTT solution was added to the wells. The media was discarded and 400 μL of DMSO was added to the wells to dissolve the formazan crystals overnight at 37 °C. Finally, the optical density (OD) value was measured at 490 nm using a spectrophotometer. The proliferation of MEFs was determined by OD values.

### 2.6. Cell Proliferation Study on Nanofibrous Scaffolds

Cell proliferation on the nanofibrous scaffolds was studied by fluorescence staining. As explained in the previous section, the same sterilization and culture process was carried out. After the incubation period, scaffolds were rinsed three times with DPBS, fixed with 4% paraformaldehyde for 10 min at room temperature and washed with DPBS (3–4 times). The cells were permeabilized with 0.3% (*v/v*) Triton (in DPBS) and 2% bovine serum albumin (BSA) for 15 min. After thorough washing in DBPS (3–4 times), the cells on the scaffolds were stained with phalloidin stain (Alexa Fluor 488, Invitrogen, Waltham, MA, USA) and incubated for 1 h. The cells were stained with DAPI (Invitrogen, Waltham, MA, USA) for 15 min to visualize their nuclei. Images were then obtained using a fluorescence microscope, at excitation wavelengths of 350 nm and 495 nm for DAPI and AlexaFluor 488, respectively.

To observe cells in FE-SEM, the scaffolds were rinsed three times with DPBS and fixed with 4% paraformaldehyde for 10 min at room temperature. Then, they were dehydrated with the gradient concentration of ethanol (50%, 70%, 80%, 95% and 100%) for 5 min each at room temperature. Finally, the scaffolds were air-dried overnight, sputtered with palladium at 18 mA for 75 s and analyzed under FE-SEM to study the morphology of attached cells on scaffolds.

### 2.7. Statistical Analysis

All data presented are expressed as mean ± standard deviations (SD). Cell viability experiments were performed in triplicate (*n* = 3) and a statistical analysis was performed using Wilcoxon test, with *p* < 0.05 indicating statistical significance.

## 3. Results and Discussion

### 3.1. Morphology and Mechanical Properties of Nanofibrous Scaffolds

**Figure 2:**
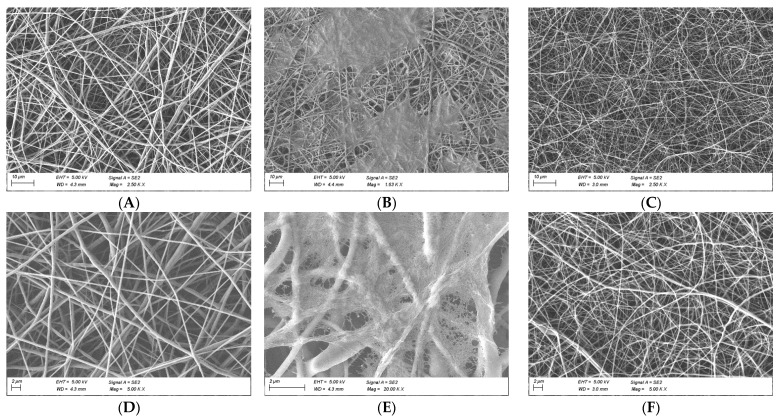
FE-SEM micrographs of electrospun scaffolds: (**A**,**D**) PCL 10%, (**B**,**E**) PCL 10% with gelatin coating, (**C**,**F**) PCL 20%: gelatin 8% (80:20). Scale bar: 10 μm (**A**–**C**); 2 μm (**D**–**F**).

The fabrication parameters of the nanofibrous scaffolds were varied until bead-free homogeneous fibers were obtained. It is well known that various parameters associated with the electrospinning method can have an impact on the morphology of the electrospun fiber [3]. Thus, we carefully chose each material by conducting a trial and error procedure and a FE-SEM analysis. For the sake of simplicity, we do not discuss the aforementioned parameters herein; instead, we present the best results in Figure 2, showing different morphologies at different scaffold compositions. Table 1 summarizes the average fiber diameter, thickness, and pore sizes of nanofibrous scaffolds. From the FE-SEM images, it is clear that the diameter of the fiber morphology decreased when gelatin was introduced as a blend, with a drop of 52%. PCL and gelatin coated PCL fibers showed similar average diameters but with a noticeable pore size decrease (57%) due to the coating (Figure 2A,B). Additionally, a 42% reduction was observed in blended PCL/gelatin scaffolds. The addition of a natural polymer like gelatin causes the size of both diameter and pore size to decrease, as shown in several studies [16,34]. Gelatin molecules have a high dielectric constant, and are likely to be charged during electrospinning. Thus, the electrospinning jet with higher gelatin content is likely to possess higher amounts of excess charge; resulting in thinner fibers [31,32].

**Figure 3:**
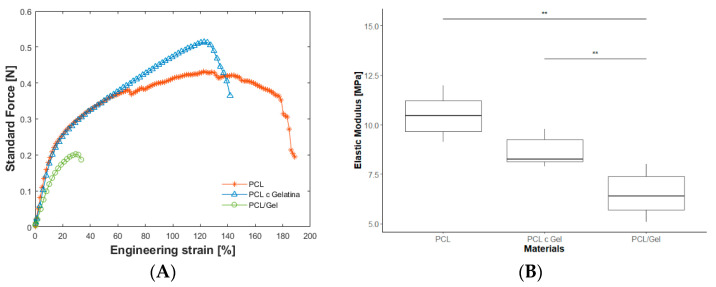
(**A**) Example of mechanical behavior of the scaffolds. (**B**) Boxplot of different materials showing the elastic modulus of PCL, PCL gelatin coating and PCL/gelatin 80:20 (** *p* < 0.05).

Regarding mechanical characterization, uniaxial tensile tests were carried out. Figure 3A shows the amount of force necessary to deform the material, whereas Figure 3B represents the Elastic Modulus for each condition. When gelatin was added as a coating on the PCL electrospun scaffold, no significant differences were observed in the stiffness except for the value of the ultimate strength. The addition of gelatin as a coating may act as a unifier between the PCL nanofibrous network, increasing the strength between the inter-fiber connections, resulting in higher ultimate strength (Figure 3A) [37,38]. Instead, after blending gelatin with PCL, the Young Modulus was reduced by 37%, which was statistically significant (*p* < 0.05). This was confirmed by the work of L. Ghasemi-Mobarakeh et al., where blending PCL with gelatin caused a reduction in mechanical strength [16] due to the weak physical properties of gelatin (which is rigid, but also fragile). It has been previously demonstrated that combining two fiber components with dissimilar mechanical properties in the same dissolution has an influence on the mechanics of the composite scaffold, which ends up displaying properties of both fiber components [33,34,35]. In addition, PCL and gelatin coated samples showed higher elongation rates than PCL/gelatin blends.

### 3.2. Fourier Transform Infrared (FT-IR) Spectroscopy

**Figure 4:**
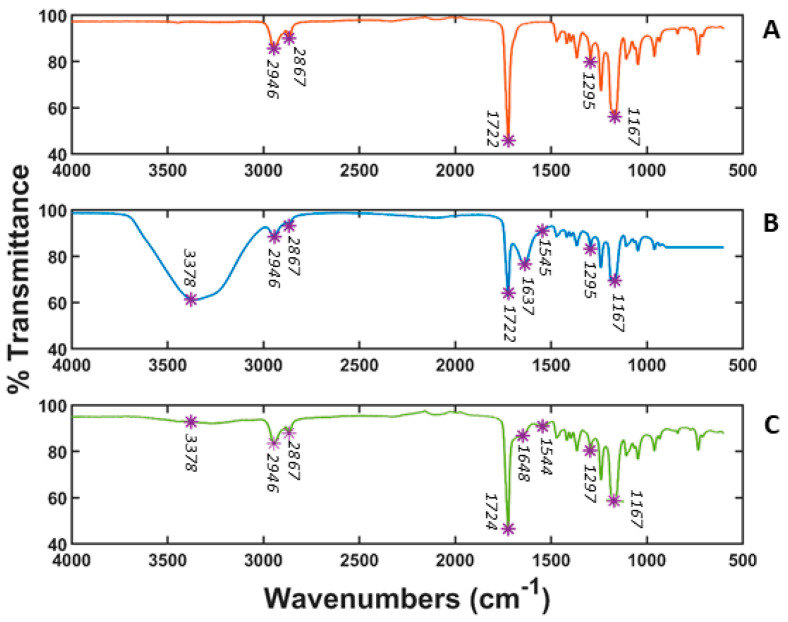
FT-IR of (**A**) PCL 10% scaffold (**B**) PCL 10% with gelatin coating and (**C**) PCL/gelatin 80:20 composite scaffold.

Figure 4 presents the FT-IT spectra of PCL, PCL gelatin coating and PCL/gelatin blend scaffolds. Several characteristic bands of PCL were observed at 2943 cm^−1^ (asymmetric CH_2_ stretching), 2867 cm^−1^ (symmetric CH_2_ stretching), 1722 cm^−1^ (carbonyl stretching), 1295 cm^−1^ (C–O and C–C stretching), and 1167 cm^−1^ (symmetric C–O–C stretching) [34,39]. The FT-IR spectrum of the gelatin coating showed bands at 3378 cm^−1^ due to N-H stretching of amide bond, C–O stretching at 1637 cm^−1^ characteristic in gelatin spectra, and N-H bending at 1545 cm^−1^. Also, the PCL main peaks exhibited lower transmittance. In the PCL/gelatin composite scaffold, all the characteristics bands of PCL and almost all those of gelatin were observed. The characteristic gelatin band at 3378 cm^−1^ was weakly detected for the PCL/gelatin scaffold. The presence of this band is much less noticeable for PCL/gelatin than for coated gelatin. This may have been due to the fact that in PCL/gelatin, the gelatin amount is lower and further way from the surface because the blending process embeds the it within the scaffold, which ultimately makes it harder to detect via FT-IR [16]. The other detected bands in PCL/gelatin were slightly shifted towards lower wave numbers. Two strong absorption bands at 1724 cm^−1^ and 1297 cm^−1^ appeared in the IR spectrum of PCL/gelatin composite scaffolds, corresponding to the characteristic bands of PCL originally situated at 1722 cm^−1^ and 1295 cm^−1^. Additionally, the distinctive amide band of gelatin at 1640 cm^−1^ showed low intensity and shifted to around 1648 cm^−1^. Another band was detected at 1544 cm^−1^, characteristic of the amide group. The shifting of original absorption bands toward lower wave numbers in the PCL/gelatin scaffolds indicated that interactions may have occurred, like hydrogen bonds between the ester groups of the PCL and the amine groups of the gelatin molecules within the scaffold [34,40]. All in all, the results indicate good interaction between PCL and gelatin in blended scaffolds, i.e., slightly different from the gelatin coated ones.

### 3.3. Cell Proliferation Studies

**Figure 5:**
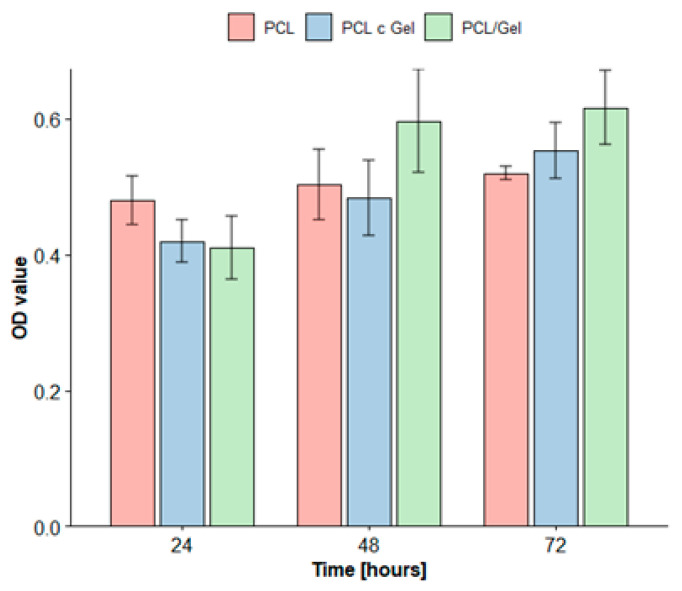
MTT results of MEF on the PCL, PCL gelatin coating and PCL/gelatin nanofibers after 24, 48 and 72 h.

Figure 5 shows the proliferation of MEFs cultured on PCL, gelatin coated PCL, and blended electrospun PCL/gelatin mats for a duration of 24, 48 and 72 h. The OD values of all the considered mats increased, which implies that all of the tested mats exhibited good biocompatibility. PCL/gelatin scaffolds showed almost a 50% growth over the 3 days, in contrast to the 8% and 31% increase observed in PCL and gelatin coated PCL scaffolds, respectively. The presence of gelatin improved cellular proliferation across all of the tested scaffolds. In particular, the highest improvement of cellular proliferation (the most statistically significant improvement (*p* ≤ 0.05)) was observed for blended scaffolds, which achieved better proliferation than PCL or gelatin coated PCL scaffolds. This was because including gelatin within the blend distributed the natural polymer throughout the entire scaffold and positively impacted cell proliferation, especially when compared to scenarios where the presence of gelatin is limited to the scaffold surface (as occurs in coating procedures). Additionally, these results indicate that the PCL, gelatin coated PCL and PCL/gelatin scaffolds did not induce any cytotoxic effects in MEF cells.

### 3.4. MEF Adhesion and Morphology Study

**Figure 6:**
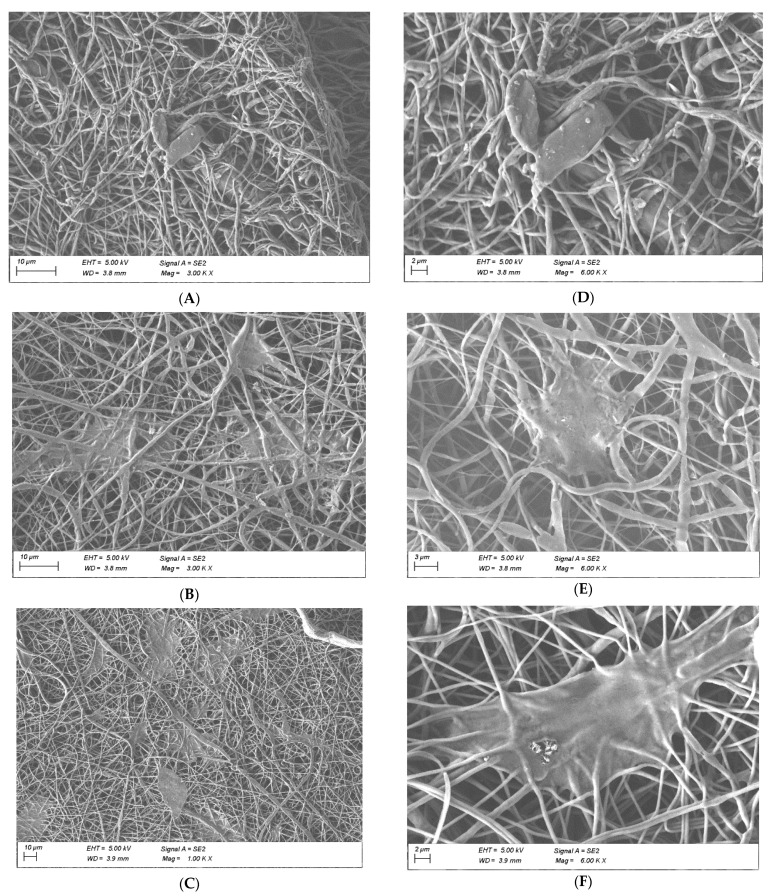
SEM images showing attachment of MEF on the surface of the scaffolds after 72 h: (**A**,**D**) PCL 10%, (**B**,**E**) PCL 10% with gelatin coating, (**C**,**F**) PCL 20%: gelatin 8% (80:20). Scale bar: 10 μm (**A**–**C**); 2 μm (**D**–**F**).

To verify the affinity of MEF on the scaffolds, a FE-SEM image analysis was performed after 72 h to evaluate cell morphology and visualize attachments (Figure 6). Cellular stretching was higher in samples containing gelatin, especially when this protein was integrated in the fibers as a blend. The further incorporation of gelatin as a blend may create longer-lasting cell attachment sites on the scaffold, which would promote cell linkage and spreading. Moreover, since gelatin was being used in an un-crosslinked state in the blended PCL/gelatin samples, it was expected that would initially absorb water, swell, and eventually leach out into the aqueous environment, i.e., cell culture media, ultimately serving two purposes; hydrophilicity for cell attachment and marginally increased pore size over time [39]. Meanwhile, the gelatin coated samples, despite yielding a better outcome than PCL scaffolds, may lose their surface functionalization faster, reducing focal linkages [23,25,41,42]. Thus, lower adhesion/stretching and infiltration rates were observed in scaffolds made up of gelatin coated PCL compared to blended PCL/gelatin scaffolds.

**Figure 7:**
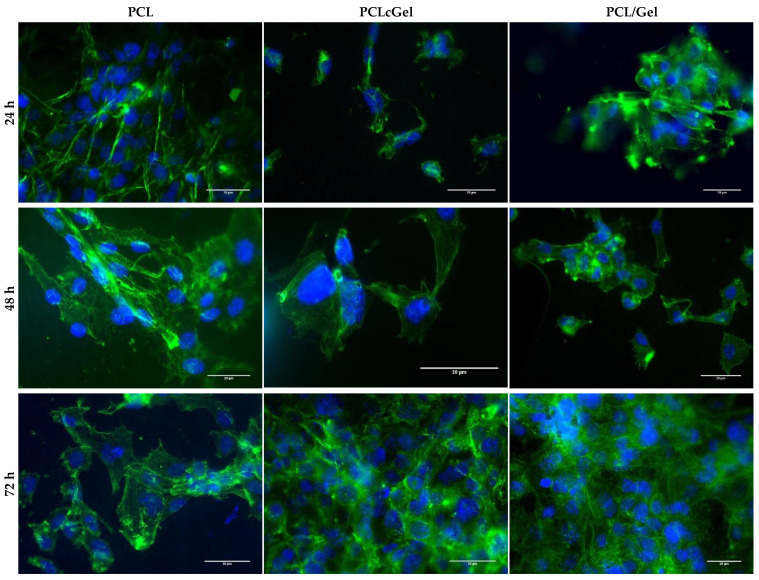
MEF proliferation study on the PCL, PCL gelatin coating and PCL/gelatin blend for 24, 48 and 72 h. (Green) Actin filaments of MEF stained with Phalloidin AlexaFluor 488. (Blue) DAPI staining the nucleus. Scale bar: 20 μm.

Figure 7 shows fluorescence images of the MEFs cultured for 24, 48, and 72 h on the three types of considered scaffolds. The number of MEFs increased from 24 to 72 h; the highest number of MEFs was observed on the blended PCL/gelatin samples. Overall, PCL based scaffolds exhibited good cellular organization after 72 h of culture.

Our results suggest an increase in cell attachment in the presence of gelatin, regardless of whether it is used as a coating or a blend [15,37]. However, blended PCL/gelatin scaffolds exhibited the best MEF response in terms of viability (Figure 5), attachment, stretching (Figure 6), and spreading (Figure 7). As shown in the results, and as generally expected, biological response improved with the increase of gelatin content. This occurred as a consequence of more integrin sites becoming available [43,44]. Our results suggest that gelatin localization (coating or blend) has a positive impact on MEF behavior, and that the presence of gelatin in a blend changes the mechanical properties and fiber morphology. Blended PCL/gelatin samples have both the lowest tensile strength and the most favorable MEF response. This is not a coincidence, since the mechanical properties of the fiber mats, an integral part of the physical microenvironment, likely played a crucial role in determining cellular fate.

The mechanical microenvironment has previously been shown to affect cell structure [45,46,47,48] and morphology, and even to modulate intracellular signaling and cell fate. Studies have reported that forces transmitted from the fiber mats can affect cell movement and change cell shape via the cytoskeleton [26,27,28,29,30,31,32,33,34,35,36,37,38,43,44,49,50,51,52]. Our results show that PCL and PCL gelatin coated samples presented higher elastic moduli and higher elongation rates compared to blended PCL/gelatin. It is likely that a more rigid network may provide a more suitable environment for cell attachment and migration. Cells may generate larger forces at focal adhesions, exerting powerful effects on the linage commitment [52]. This was evident in our experimental observations, where improvements in cell stretch and adhesion were found for blended PCL/gelatin samples. The adhesion and morphology of the MEFs might be compromised in samples with no gelatin content. This happens because of their higher Young Modulus, higher elongation rates, and the reduction on integrin adhesions [25,53].

## 4. Conclusions

We studied the ways in which gelatin coalescence (coating or blend) enhances the performance of PCL scaffolds in terms of their mechanical properties and cellular response. We also provided a thorough analysis of the differences between using gelatin coated PCL and blended PCL/gelatin scaffolds. PCL and gelatin coated PCL scaffolds showed higher elongation rates and stiffness than the blended PCL/gelatin samples, which had better MEF responses in terms of cell attachment and spreading. Furthermore, our results also indicated that the fiber diameter distribution and pore size were modulated after blending with gelatin. Blended gelatin not only positively influences cellular response, but also improves the mechanical and morphological properties of the fibrous network. Our results suggest that PCL/gelatin blends enhance the performance of polymeric scaffolds, both in cellular and mechanical aspects. All in all, these outcomes provide evidence of the superiority of blended PCL/gelatin scaffolds over their PCL and PCL gelatin coated counterparts under certain circumstances, such as would healing [32,33].

This work suggests that this methodology could be extended to create other natural and synthetic polymer blended combinations, and that similar results may be expected. The challenges of using polymeric blends usually lie in the creation of stable solutions and the compatibility of the chosen materials. The incorporation of blends in the development of scaffolds for tissue engineering applications undoubtedly shows potential and more promising results than coating protocols for further improvements in scaffold designs.

## Figures and Tables

**Table 1:** Fiber diameter, thickness and pore size measurements of PCL, PCL coated by gelatin and PCL/gelatin.

Substrate	Thickness (μm)	Fiber Diameter (nm)	Pore Size (μm^2^)
PCL	20.25 ± 1.25	702 ± 268	2.1 ± 3.5
PCL coated with gelatin	20.75 ±1.70	804 ± 150	0.9 ± 2.9
PCL/gelatin (80:20)	19.65 ± 7.68	337 ± 106	1.2 ± 2

## Data Availability

Data is contained within the article.
